# Corrosive Sublimate for Hardening the Brain for Anatomical Study

**Published:** 1888-08

**Authors:** 


Corrosive Sublimate for Hardening the Brain for Anatomical Study.—Dionridoff, of St. Petersburg, has employed a 7 per cent. solution of corrosive sublimate to harden the tissues of the brain and nervous system. Small pieces of tissue were placed in this solution from seven to nine days, after which they were put in alcohol, 50, 70, and 96 per cent., for twenty-four hours. This method of hardening preserves the contour of the cells; the tissue so hardened cuts and stains well, and is easily preserved.—*Fortschritte der Medicin*, April 15, 1888.

